# Imbalance of Pro-Oxidant and Anti-Oxidant Biomarkers in Patients with Keratoconus

**DOI:** 10.3390/antiox15030287

**Published:** 2026-02-26

**Authors:** Ariadna Patricia Nicula, Cristina Ariadna Nicula, Dorin Nicula, Karin Ursula Horvath, Camelia Manuela Mîrza, Paul Mihai Boarescu, Sorana D. Bolboacă, Adriana-Elena Bulboacă

**Affiliations:** 1 Medical Informatics and Biostatistics, Department 11—Medical Education, Faculty of Medicine, “Iuliu Hațieganu” University of Medicine and Pharmacy, Louis Pasteur Str., No. 6, 400349 Cluj-Napoca, Romania; nicula.ariadnapatricia@elearn.umfcluj.ro; 2 Oculens Clinic, Calea Turzii, No. 134, 400347 Cluj-Napoca, Romania; cristina.nicula@umfcluj.ro (C.A.N.);; 3 Medical Specialties, Department 1—Maxillo-Facial Surgery and Radiology, Faculty of Dental Medicine, “Iuliu Hațieganu” University of Medicine and Pharmacy, Clinicilor Str., No. 3-5, 400006 Cluj-Napoca, Romania; 4 Department of Ophthalmology, “George Emil Palade” University of Medicine and Pharmacy Science and Technology, Márton Áron Str., No. 26, 540058 Târgu Mureș, Romania; 5 Pathophysiology, Department 1—Morpho-Functional Sciences, Faculty of Medicine, “Iuliu Hațieganu” University of Medicine and Pharmacy, Victor Babeş Str., No. 2-4, 400012 Cluj-Napoca, Romania; 6 Department of Medical-Surgical and Complementary Sciences, Faculty of Medicine and Biological Sciences, “Ștefan cel Mare” University of Suceava, Universității Str., No. 13, 720229 Suceava, Romania; paul.boarescu@usm.ro

**Keywords:** oxidative stress, keratoconus (KCN), nitric oxide, total oxidant status, total anti-oxidant status, biomarker

## Abstract

Aim: Our study aimed to evaluate serum oxidative stress-related biomarkers (three pro-oxidants: total oxidant status (TOS), indirect assessment of nitric oxide synthesis (NOx), and malondialdehyde (MDA), and three anti-oxidants: total anti-oxidant capacity (TAC), catalase (CAT), and thiols) in patients with keratoconus (KCN). Methods: We conducted a single-center, cross-sectional study with the prospective enrollment of adult patients attending an outpatient ophthalmology clinic between 1 January 2024, and 1 September 2025. The diagnosis of KCN was established based on the clinical signs and two or more aberrant Pentacam parameters. Results: We evaluated 44 subjects with KCN (median age, 26 years) and 38 without KCN (median age, 28 years), with similar age and sex distributions (*p*-values > 0.09). All evaluated biomarkers showed statistically significant differences between subjects with KCN and those without KCN (*p*-values < 0.001), with higher serum TOS, NOx, MDA, CAT, and oxidative stress index levels and lower levels of TACs and thiols in subjects with KCN than in those without KCN. Conclusions: Our findings indicate a systemic imbalance between pro-oxidant and anti-oxidant biomarkers in subjects with keratoconus.

## 1. Introduction

Keratoconus (KCN) is a bilateral, asymmetric, ectatic corneal dystrophy that develops during puberty, with rapid evolution between 10 and 20 years of age [1,2]. The shape of the cornea changes in evolution in the paracentral and central parts, giving rise to irregular astigmatism, myopia, decreased visual acuity, and thinning of the corneal tissue [3]. Keratoconus is a multifactorial disease with variable progression between eyes. The etiology and pathogenesis of KCN remain incompletely elucidated. Several factors, including genetics, family aggregation, eye rubbing, atopy, sex hormones, prolactin-inducible proteins, and inflammation, are implicated in KCN pathogenesis [4,5]. Oxidative stress is a significant risk factor and contributor to KCN, causing an imbalance between damaging oxidants and anti-oxidant molecules [6,7,8,9,10]. The accumulation of reactive oxygen species (ROS) resulting from exposure of the cornea to ultraviolet (UV) radiation can damage cells by reacting with proteins, deoxyribonucleic acid (DNA), and membrane phospholipids [11,12]. Reactive oxygen species generated after UV exposure stimulate lipid peroxidation, which results in the production of reactive aldehydes such as malondialdehyde (MDA) and 4-hydroxy-2-nonenal (HNE). In keratoconic corneas, MDA and HNE can damage cellular biomolecules, affecting keratocytes and leading to corneal thinning and protrusion [11].

Under physiological conditions, cellular redox homeostasis is preserved; however, there is an imbalance between reactive oxygen species (ROS) and anti-oxidant responses. Because of its location, the cornea is permanently exposed to sunlight, including ultraviolet (UV) radiation, metabolic activity, and atmospheric oxygen, mainly dioxygen, which produces ROS [13]. The healthy cornea presents several anti-oxidative mechanisms that can be endogenous enzymatic (such as superoxide dismutase (SOD), catalase (CAT), glutathione peroxidase (GP)), non-enzymatic (e.g., glutathione, melatonin, bilirubin), and exogenous (e.g., vitamin C, vitamin E, carotenoids) sources to eliminate ROS [10,11]. The role of nitric oxide (NO) in the pathogenesis of KCN is complex, as it induces corneal damage at high concentrations and with low values in normal physiological processes. Nitric oxide is a gaseous molecule that diffuses across cell membranes and plays a role in vasodilation, neurotransmission, and cytotoxicity [14]. Nitro-oxidative stress results from the excessive production of reactive nitrogen species (RNS), which consist of NOx and peroxynitrite anions (ONOO−). Suppression of RNS production is associated with anti-inflammatory activity, and NOx is a sensitive biomarker for anti-inflammatory effects [15]. Inflammation and oxidative stress are interrelated: inflammation induces oxidative injury, and oxidative stress triggers inflammation. Studies have shown that both oxidative and nitro-oxidative stress or a decrease in defense mechanisms (anti-oxidant systems) can provoke the inflammatory process, which is an important part of KCN pathogenesis [4,5]. Excessive NOx can lead to the activation of matrix metalloproteinases (MMPs), which are enzymes that degrade the extracellular matrix of the cornea and contribute to corneal tissue damage [4,5]. Patients with KCN have abnormal levels of anti-oxidant enzymes [16], increased mitochondrial DNA damage [17,18,19], accumulation of cytotoxic byproducts from lipid peroxidation and NO pathways, and increased levels of proinflammatory cytokines in their tears and corneas [20].

Although several studies have implicated systemic oxidative stress in the pathogenesis of keratoconus (KCN) [21,22,23,24,25,26,27], data from Eastern European populations and specifically from the Romanian population are currently lacking. Moreover, while the total oxidant status (TOS) and total anti-oxidant capacity (TAC) are widely used to characterize oxidative balance [28,29], comprehensive evaluations integrating both biomarkers in KCN remain limited. To address these gaps, the present study aimed to characterize systemic oxidative stress in a Romanian cohort of individuals with KCN by assessing the serum NOx (an indirect marker of NO synthesis), TOS, TAC, thiols, catalase (CAT), and malondialdehyde (MDA).

## 2. Materials and Methods

This study was conducted at the Oculens Clinic in Cluj-Napoca, Romania, in accordance with the latest update (2024) of the Declaration of Helsinki. This study was approved by the Oculens Ethical Committee (approval no. 14/1, December 2023). All procedures complied with ethical standards, ensuring the protection of the participants’ rights, safety, and well-being. All the participants provided written informed consent.

### 2.1. Design Settings

A single-center, cross-sectional, observational study was conducted among patients referred to an outpatient clinic for eye examination, diagnosis, and/or treatment. The enrollment was prospective, from 1 January 2024, to 1 September 2025.

At the time of enrollment, subjects were (1) aged 18 years or older, (2) provided consent for corneal tomography and biomechanical measurements, and (3) agreed to provide a blood sample for measurement of evaluated biomarkers. Subjects who self-reported previous corneal surgeries (cross-linking procedure, intracorneal ring implantation, corneal grafts), systemic inflammatory/oxidative disease, topical/systemic anti-oxidants, steroid therapy, vitamin supplements, pregnancy, or malignancy were not eligible for enrollment.

### 2.2. Ocular Examination and Keratoconus Diagnosis

All patients underwent a comprehensive ophthalmological examination performed by two ophthalmologists with similar experience (~18 years) following the clinic’s standard protocol, which included uncorrected visual acuity (UCVA) and corrected distance visual acuity (CDVA) using the Snellen charts and subsequently converted to logMar charts for statistical keratometry and ocular refraction (Topcon KR 8900 autorefractor-keratometer, Topcon Corporation, Tokyo, Japan), measurement of intraocular pressure with the applanotonometer (Haag-Streit AT 900 applanation tonometer, Haag-Streit AG, Köniz, Switzerland), slit-lamp examination (Haag-Streit BX 900 slit lamp, Haag-Streit AG, Köniz, Switzerland), and dilated fundus examination with the non-contact +90 D Volk lens. Before the exam of the fundus, the corneal tomography (Oculus Pentacam, Oculus Optikgerate GmbH, Wetzlar, Germany) was performed by an experienced single assistant, and the biomechanical measurements were made with Corvis^R^ ST (Oculus Corvis, Oculus Optikgerate GmbH, Wetzlar, Germany software version 1.5r1902), which incorporates the Pentacam parameters with the biomechanical data [20] and records corneal deformation responses after the application of a standardized air puff [30].

The diagnosis of KCN was based on the typical signs revealed by slit-lamp examination (Vogt’s striae, Fleisher rings, and Munson) [3] and the presence of scissor reflex and irregular astigmatism on retinoscopy. Topographic parameters, such as zonal maximum keratometry in a 3 mm zone around the steepest point (zonal Kmax-3 mm), K max > 48 D [3], ART-max < 339 [31], IS-value  >  1.4 [32], Belin–Ambrósio deviation index (BAD-D)   >  1.6 [33], minimum corneal thickness, and a posterior elevation map indicated the presence of KCN. The diagnosis of KCN was established based on the clinical signs and two or more aberrant Pentacam parameters. The severity of KCN was classified based on the ABCD grading system [34]. Given the high variability in severity assessment using the ABCD grading system, we classified the severity of KCN based on Kmax as mild (Kmax ≤ 47 D, D is Diopters), moderate (Kmax higher than 47 and less than or equal to 52 D), and severe (Kmax > 52 D).

### 2.3. Measurement of Evaluated Biomarkers

Five milliliters of peripheral venous blood was obtained from all participants in the treatment room on the same day as the clinical visit; however, the fasting status at the time of sampling was not routinely recorded. Samples were collected in plain red-top tubes without additives, allowed to clot, and subsequently centrifuged at 5000 rpm for 10 min. The separated serum was carefully aliquoted and stored at −80 °C until biochemical analyses were performed.

The methods used to determine the pro-oxidant (TOS, NOx, and MDA) and anti-oxidant (TAC, CAT, and thiols) biomarkers are summarized in Table 1. All spectrophotometric measurements were performed by the same technician, blinded to the pathology, using a Jasco V-350 UV–VIS spectrophotometer (Jasco International Co., Ltd., Tokyo, Japan).

Age and sex data were obtained from participants upon informed consent signing.

### 2.4. Statistical Analysis

Three composite indices derived from the measured biomarkers were calculated: oxidative stress index (OSi) = TOS/TAC [40], MDA/TAC ratio, and MDA/TOS ratio. High values of these indices indicate increased oxidative stress or imbalance.

An exploratory statistical analysis was performed to evaluate the differences in the oxidative stress biomarkers between individuals with and without KCN and across the KCN severity classes. Categorical data were summarized as counts and percentages, and group comparisons were conducted using the chi-squared test. Continuous variables were first assessed for normality within each group using the Shapiro–Wilk test. Normally distributed continuous variables are reported as means (standard deviations) and were compared between groups using the independent sample Student’s *t*-test (with and without KCN) or one-way ANOVA (KCN severity classes). Non-normally distributed variables were summarized as medians [Q1–Q3] and compared using the Mann–Whitney U test (with and without KCN) or the Kruskal–Wallis test (KCN severity classes). Correlation analyses were conducted for each group of patients (with and without KCN) using Pearson’s correlation for normally distributed variables and Spearman’s rank correlation for non-normally distributed variables. The rank–biserial correlation coefficient was the measure of the effect size reported when the Mann–Whitney test indicated a small effect for |r|≥ 0.1, a medium effect for |r|≥ 0.28, and a large effect for |r|≥ 0.43 [41]. The effect size associated with Student’s *t*-test was Cohen’s d, with a small effect for d = 0.2, a medium effect for d = 0.5, and a large effect for d ≥ 0.8 [42]. The epsilon-squared (ε^2^) statistic was reported as an effect size measure in the comparison among the KCN severity groups, considering negligible-to-weak effects for values less than 0.06, moderate effects for values higher than 0.06 and smaller than 0.14, and large effects for values higher than or equal to 0.14 [41].

Statistical analysis was performed using Statistica software (v. 13.1, TIBCO, Palo Alto, CA, USA), and graphical representations were generated using JAMOVI (v. 2.6.26.0). Statistical significance was defined as a two-sided *p*-value smaller than 0.05.

## 3. Results

### 3.1. Between-Group Analysis: Keratoconus vs. Controls

Eighty-two patients, aged 18–38 years, 44 with KCN and 38 controls, were evaluated. Most participants were men (*n* = 51, 62.2%), with no statistically significant differences in sex and age between the groups (Table 2).

According to the Belin ABCD keratoconus staging system, the variability was high in our cohort. The top three most frequent stages were A4B4C2D1 (six cases), A2B3C1D2 and A0B2C0D1 (each with three cases), and A0B2C2D1 (three cases).

**Figure 1:**
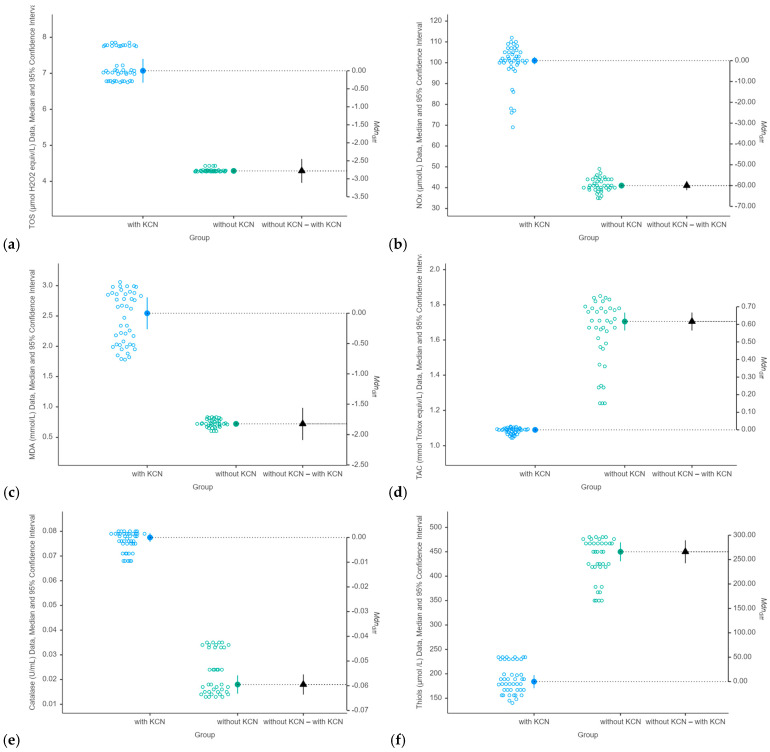
Distribution of evaluated biomarkers by group. Pro-oxidant markers: (**a**) TOS—total oxidant status, (**b**) NOx—indirect assessment of nitric oxide synthesis, and (**c**) MDA—malondialdehyde; anti-oxidant markers: (**d**) TAC—total anti-oxidant capacity, (**e**) CAT—catalase, and (**f**) thiols. The transparent circles denote raw data; the colored circles indicate the median values, with 95% confidence intervals shown by the lines; the triangles indicate the median differences (Mdn_diff_) with associated 95% confidence intervals.

Individuals with KCN exhibited higher pro-oxidant marker levels compared to those without KCN, with the differences reaching statistical significance (Table 2, Figure 1). Catalase followed a similar pattern, whereas both the TACs and thiols showed lower levels in individuals with KCN than in those without KCN (Table 2, Figure 1). Elevated values of the evaluated ratios (OSi, MDA/TAC, and MDA/TOS) were observed in the subjects with KCN (Table 2), reflecting increased oxidative stress.

**Figure 2:**
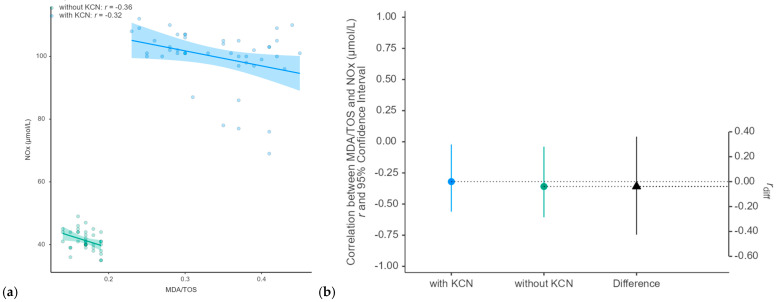
Correlation analysis showing (**a**) scatter plot and (**b**) estimation in association analysis. The transparent blue circles denote raw data for subjects with KCN, while the transparent green circles denote patients without KCN; the colored circles indicate the values of the estimates, with 95% confidence intervals shown by the lines; the triangles indicate the correlation coefficient differences (r_diff_) with associated 95% confidence intervals. The colored belt is the 95% confidence interval.

A statistically significant indirect monotonic association was observed between the NOx and MDA/TOS in individuals with KCN (Spearman’s correlation coefficient, *p*-value = 0.038) and without KCN (*p*-value = 0.031) (Figure 2). In individuals without KCN, thiols showed a statistically significant inverse monotonic association with catalase (Spearman’s correlation coefficient: −0.50, *p*-value = 0.001), while NOx was significantly correlated with the OSi (Spearman’s correlation coefficient: 0.359, *p*-value = 0.027). No statistically significant associations were observed between age and the evaluated biomarkers across the groups (*p*-values > 0.08).

### 3.2. Severity-Based Analysis Within the Keratoconus Group

Four patients (one female and three males) had mild KCN with a balance of moderate and severe KCN among women (6/13 for each severity class), and more male patients had severe KCN (18/31, 58.1%). No statistically significant association was identified between sex and the KCN severity (Fisher’s exact test: *p*-value = 0.7804). The KCN severity had no statistically significant effect on the evaluated biomarkers (Table 3).

## 4. Discussion

Our results demonstrated that keratoconus is associated with systemic oxidative stress. The consistent and statistically significant differences across all evaluated biomarkers (Table 2 and Figure 1) provide strong evidence that patients with KCN exhibit oxidative imbalance. The evaluated biomarkers revealed two key components: increased oxidant production and lipid peroxidation and a dysregulated anti-oxidant capacity. Elevated serum levels of TOS, NOx, and MDA directly indicate elevated reactive species levels and subsequent lipid oxidative damage. The decrease in the serum total anti-oxidant capacity (TAC) and thiol levels suggests depletion of the body’s general anti-oxidant reserves. The increase in CAT activity may represent a compensatory upregulation of specific anti-oxidant enzymes in response to sustained oxidative stress. A statistically significant inverse correlation between NOx and the MDA/TOS ratio was observed in all individuals with and without KCN (Figure 2). The correlation between NOx and the oxidative stress index (OSi) in individuals without KCN suggests that this relationship is disrupted in the disease state. The absence of statistically significant associations between age and any evaluated biomarker in either group suggests that the observed differences in the oxidative stress profiles are unlikely to be explained by age alone and may be attributed to keratoconus.

Oxidative stress, an imbalance between reactive oxygen species (ROS) production and anti-oxidant defenses, has been implicated in the development and progression of KCN. Evidence suggests that oxidative stress can damage cellular components, potentially contributing to the characteristic thinning and bowing of the cornea, a major hallmark of KCN [16,27]. Previous studies highlighted that local oxidative stress markers were increased, and that the anti-oxidant capacity and glutathione concentration in KCN corneas were decreased compared to healthy corneas [8,10,26,27,43]. The presence of pro-inflammatory cytokines (IL-1, IL-6, TNF-α, and TGF-β), degradation of collagens by metalloproteases (MMP-1, MMP-3, and MMP-9), and lysyl oxidase (LOX) in cornea tissues and tears highlight the idea that KCN is an inflammatory disease [4,5,44,45,46,47,48,49,50,51,52,53,54,55]. Mazzota et al. [56,57] showed that the Epi-off accelerated cross-linking technique (ACXL) may stabilize ectasia, improve visual and ocular surface outcomes, and significantly lower tear MMP-9 levels. In addition, studies have reported the anti-inflammatory properties of vitamin D in ocular diseases [58].

Our findings showed that individuals with KCN had elevated pro-oxidant marker levels, including TOS, NOx, and MDA, compared to individuals without KCN (Table 2, Figure 1), with differences reaching statistical significance (*p*-values < 0.001). Catalase followed a similar pattern, whereas both the TAC and thiols showed lower levels in individuals with KCN than in those without KCN (Table 2, Figure 1, *p*-values < 0.001). Moreover, the evaluated ratios (OSI, MDA/TAC, and MDA/TOS) were elevated in KCN individuals, indicating increased oxidative stress. The relationship between oxidative stress and anti-oxidant defenses, particularly the role of catalase in keratoconus, is complex [59]. The high levels of catalase observed in our cohort are consistent with the findings reported in the scientific literature [60] and may reflect a compensatory response to oxidative stress despite the depletion of anti-oxidant reserves. These findings suggest that although catalase is upregulated in response to oxidative stress, the overall anti-oxidant capacity is compromised, potentially contributing to disease progression.

Our results are similar to those reported by Toprak et al. [27], in which the serum total TOS and OSi levels were statistically significant higher in individuals with KCN than in those without KCN, although there was no significant difference in the TACs between the two groups. Moreover, Arnal et al. [11] found that total nitrites and lipid peroxidation were elevated in the corneas of individuals with KCN when compared with the controls. Similarly, clinical studies have found that tears from individuals with KCN have lower levels of glutathione (an anti-oxidant that mitigates oxidative stress), higher proteolytic activity, and overexpression of several matrix metalloproteinases compared with healthy controls [43,48]. Moreover, Behndig et al. [9] showed that extracellular superoxide dismutase levels were significantly decreased in keratoconic corneas. Furthermore, some studies suggest that oxidative stress induces the activation of tissue proteinases and degradation of proteinase inhibitors, leading to progressive corneal thinning, a major hallmark of KCN [22,61]. Kenney et al. [8] demonstrated reduced levels of the tissue inhibitor of matrix metalloproteinase 1 and increased levels of cathepsin V/L2 (a cysteine proteinase) and catalase in keratoconic corneas.

Our study demonstrated a statistically significant, monotonic, and indirect association between the NOx and MDA/TOS in individuals with KCN as well as in those without KCN (Figure 2). These findings illustrate the interplay between anti-oxidants and oxidative stress markers in healthy individuals and highlight the biological mechanisms that maintain homeostasis and prevent oxidative damage. Individuals with KCN are expected to exhibit an imbalance in oxidative stress, whereas those without KCN are expected to have normal levels of oxidative stress markers. Imbalances in oxidative stress and/or systemic inflammation biomarkers can be linked to various situations, such as stress [62], obesity [63], sleep quality [64], smoking [65,66], diabetes [67], hepatotoxicity injuries [68], physical activity [69,70], and water intake [71]. No statistically significant associations were observed between age and the evaluated biomarkers across all groups. Similarly, Toprak et al. [27] revealed that the serum TOSs and OSis were significantly elevated in patients with KCN compared to controls, whereas the TACs showed no significant difference between the groups. These results indicate that patients with KCN are exposed to potent oxidative stress, with redox balance shifting toward oxidation, whereas anti-oxidant defenses remain unchanged.

The distribution of disease severity within the KCN group was uneven, comprising four mild, 16 moderate, and 24 severe cases, resulting in a relatively small number of participants with mild disease. Although subjects with mild KCN tended to be older, the age difference between individuals with moderate and severe KCN was not statistically significant (Table 3). When biomarker levels were examined according to severity, a numerical trend toward lower values in severe cases was observed for the TOC, NOx, catalase, and thiols. Our findings align with those reported by Kilic et al. [22], who observed no statistically significant differences in the TAC and TOS levels among KCN severity groups. The lack of statistical significance may be attributed to limited statistical power stemming from the small number of mild cases and the restricted overall sample size. The presence of trends across the severity strata underscores the need for future research with larger cohorts and a balanced severity distribution to conclusively evaluate the role of oxidative stress in patients with KCN.

### Strengths and Limitations

To the best of our knowledge, this study is the first in Romania to evaluate serum oxidative stress biomarkers in patients with KCN by measuring serum NOx, TOS, TAC, MDA, thiol, and catalase levels. The evaluation of an outpatient population following a standardized protocol at a single center, which enabled consistency in the clinical assessment and biomarker measurements, is a strength of our study. The applied design enhances internal validity and enables assessment of oxidative stress in a real-world ambulatory setting, providing clinically relevant insights that may inform routine patient management.

Our study has several limitations. First, although standardized outpatient care within a single center represents a methodological strength, it may also reflect local clinical practices and demographic characteristics, thereby limiting the external validity and generalizability of the findings to other populations and healthcare settings. Second, recruiting participants from a single outpatient clinic may introduce a selection bias, as these individuals are likely to be more health-conscious, compliant, and aware of their disease. Third, the evaluated biomarkers were not disease-specific, and no adjustment for potential confounding factors was made, considering that multiple factors are associated with an increase in oxidative stress (e.g., stress, ischemia, bleeding, infection, radioactivity, medications, long-term metabolic diseases, sun exposure, smoking, physical activity, diet, supplement use, allergic disease, eye rubbing behavior, and the aging process [72,73,74,75]). Fourth, the oxidative stress markers evaluated were measured at a single time point and may be affected by transient factors, such as diet, physical activity, stress, smoking status, medications, supplements, and comorbidities. Furthermore, eligibility for participation was based on the self-report of no supplement use; therefore, the possibility of unrecognized anti-oxidant intake cannot be entirely excluded. Therefore, the absence of a documented washout period or more rigorous screening may represent a potential source of residual confounding. Consequently, prospective follow-up studies should evaluate biomarkers with more rigorous screening and, in cases of supplement intake, include a washout period to exclude potential sources of residual confounding factors. Future investigations conducted across multiple centers and healthcare settings, including both outpatient and inpatient populations, with stratification by disease severity, repeated biomarker assessments over time, and careful consideration of lifestyle- and treatment-related factors would provide more robust and generalizable evidence. Additionally, the evaluation of oxidative stress biomarkers in tear serum and fluid may represent a promising noninvasive approach for disease assessment and monitoring [76,77]. Larger multicenter longitudinal studies are needed to confirm and extend our findings and provide input data for the early detection of artificial intelligence tools.

## 5. Conclusions

Our results show that individuals with keratoconus exhibit a marked imbalance in systemic oxidative stress, characterized by higher serum levels of total oxidant status, nitric oxide metabolites, and malondialdehyde, along with an elevated oxidative stress index, compared with individuals without keratoconus. These findings are consistent with greater oxidative damage and increased lipid peroxidation in individuals with keratoconus. Concurrently, subjects with keratoconus demonstrated significantly lower systemic anti-oxidant defenses, reflected by reduced total anti-oxidant capacity and thiol levels, indicating a diminished anti-oxidant profile. The observed higher levels of catalase activity may reflect an adaptive response to increased oxidative stress, rather than the restoration of effective redox balance. Overall, the reported findings suggest that oxidative stress-related biomarkers may be useful for disease characterization and monitoring; however, these observations should be interpreted with caution because the analyses were not adjusted for potential confounding factors, including smoking status, body mass index, metabolic diseases, physical activity, diet, supplement use, allergic disease, eye rubbing behavior, or other factors that possibly affect systemic oxidative status. Future longitudinal and multicenter studies are needed to clarify causality, assess associations with disease severity and progression, and further investigate the potential role of anti-oxidant-based therapeutic strategies in keratoconus.

## Figures and Tables

**Table 1:** Laboratory methods for evaluating biomarkers.

Biomarker, Units of Measurement	Abbreviation	Method [Ref.]
Total Oxidant Status, μmol H_2_O_2_ equiv/L	TOS	Colorimetric automated method based on oxidation of ferrous to ferric ion [28]
Total Anti-Oxidant Capacity, mmol Trolox equiv/L	TAC	Erel method based on free radical reactions initiated via hydroxyl radicals (Fenton reaction) [35]
Indirect Assessment of Nitric Oxide Synthesis, μmol/L	NOx	Reduction of nitrate by vanadium (III) followed by acidic Griess reaction (Miranda et al. [36])
Malondialdehyde, mmol/L	MDA	Thiobarbituric acid reactive substances (TBARS) assay following Mitev et al. [37]
Catalase, U/mL	CAT	Spectrophotometric measurement [38]
Thiols, μmol /L	Thiols	Spectrophotometric assay using DTNB (Ellman’s reagent) [39]

**Table 2:** Comparison of demographics and evaluated biomarkers between groups.

Variable	With KCN (*n* = 44)	Without KCN (*n* = 38)	Stat. (*p*-Value)	Effect Size
Age, years ^a^	26 [19.8 to 32]	28 [22.3 to 30]	−0.7 (0.465)	0.09
Sex ^b^			2.8 (0.097)	
Male	31 (70.5)	20 (52.6)		
Female	13 (29.5)	18 (47.4)		
TOS, μmol H_2_O_2_ equiv/L ^a^	7.07 [6.8 to 7.8]	4.29 [4.28 to 4.30]	7.8 (<0.001)	−0.99
NOx, μmol/L ^c^	99.8 (9.5)	41.3 (3.2)	36.2 (<0.001)	8.02
MDA, mmol/L ^c^	2.46 (0.43)	0.73 (0.07)	24.6 (<0.001)	5.44
TAC, mmol Trolox equiv/L ^a^	1.089 [1.076 to 1.095]	1.705 [1.565 to 1.78]	−7.8 (<0.001)	0.99
Catalase, U/mL ^a^	0.078 [0.075 to 0.079]	0.018 [0.015 to 0.033]	7.8 (<0.001)	−0.99
Thiols, μmol/L ^a^	184 [167 to 230]	450 [419 to 467]	−7.8 (<0.001)	0.99
OSi ^a^	6.5 [6.3 to 7.1]	2.5 [2.4 to 2.8]	7.8 (<0.001)	−0.99
MDA/TAC ^c^	2.21 (0.52)	0.45 (0.07)	28.0 (<0.001)	6.19
MDA/TOS ^c^	0.4 (0.23)	0.17 (0.02)	16.3 (<0.001)	3.60

^a^ Median [Q1 to Q3], where Q indicates the value of the quartile; a comparison is made with the Mann–Whitney test and rank–biserial correlation coefficient reported as a measure of the effect size; ^b^ number (%), comparison with chi-squared test; ^c^ arithmetic mean (standard deviation); comparison made with Student’s *t*-test for independent samples, and Cohen’s d reported as measure of effect size; TOS—total oxidant status, NOx—indirect assessment of nitric oxide synthesis, MDA—malondialdehyde, TAC—total anti-oxidant capacity, OSi—oxidative stress index; effect size is represented by rank–biserial correlation coefficient when Mann–Whitney test was applied, and by Cohen’s d when Student’s *t*-test was used to compare the groups.

**Table 3:** Serum values of evaluated biomarkers by KCN severity group.

Variable	KCN Severity			Stat. (*p*-Value)	ε^2^
	Mild (*n* = 4)	Moderate (*n* = 16)	Severe (*n* = 24)		
Age, years	30 [28 to 33.5] {28 to 38}	24 [19.75 to 29] {18 to 36}	25 [18.75 to 30.5] {18 to 36}	3.4 (0.1878)	0.08
TOS, μmol H_2_O_2_ equiv/L	7.4 [6.963 to 7.78] {6.8 to 7.8}	7.08 [7.01 to 7.765] {6.75 to 7.85}	7.02 [6.78 to 7.75] {6.75 to 7.85}	2.0 (0.3688)	0.05
NOx, μmol/L	107.5 [105.5 to 109] {101 to 112}	103 [96.8 to 105.3] {69 to 110}	101 [99.8 to 102.3] {76 to 109}	5.1 (0.0784)	0.12
MDA, mmol/L	1.965 [1.833 to 2.125] {1.78 to 2.26}	2.715 [2.208 to 2.92] {1.95 to 2.99}	2.64 [2.0125 to 2.835] {1.79 to 3.06}	5.9 (0.0526)	0.14
TAC, mmol Trolox equiv/L	1.0895 [1.086 to 1.091] {1.078 to 1.092}	1.0925 [1.085 to 1.099] {1.056 to 1.106}	1.087 [1.065 to 1.094] {1.045 to 1.108}	3.7 (0.1541)	0.09
Catalase, U/mL	0.076 [0.074 to 0.077] {0.071 to 0.08}	0.079 [0.076 to 0.079] {0.071 to 0.08}	0.076 [0.071 to 0.079] {0.068 to 0.08}	2.7 (0.2536)	0.06
Thiols, μmol/L	200.5 [164.25 to 234] {156 to 234}	184 [167 to 198.25] {140.3 to 234}	184 [167 to 230] {145 to 234}	0.3 (0.8745)	0.01
OSi	6.78 [6.41 to 7.13] {6.3 to 7.14}	6.46 [6.38 to 7.16] {6.15 to 7.35}	6.47 [6.39 to 7.11] {6.17 to 7.38}	<0.1 (0.9989)	<0.01
MDA/TAC	1.805 [1.683 to 1.958] {1.63 to 2.1}	2.475 [2.058 to 2.713] {1.78 to 2.74}	2.435 [1.89 to 2.613] {1.64 to 2.9}	5.6 (0.0605)	0.13
MDA/TOS	0.27 [0.24 to 0.31] {0.23 to 0.33}	0.36 [0.3 to 0.41] {0.26 to 0.44}	0.36 [0.29 to 0.39] {0.24 to 0.45}	4.6 (0.0987)	0.11

Data are summarized as medians [Q1 to Q3], where Q indicates the value of the quartile and {minimum to maximum}; comparison was made with the Friedman test; TOS—total oxidant status, NOx—indirect assessment of nitric oxide synthesis, MDA—malondialdehyde, TAC—total anti-oxidant capacity, and OSi—oxidative stress index; ε^2^—epsilon-squared statistic.

## Data Availability

Data supporting the findings of this study are available from the first author upon reasonable request. The data are part of an ongoing PhD research project and are therefore not publicly available at this time.
